# Prevalence, types and disclosure of complementary and alternative medicine (CAM) use among chronic kidney disease (CKD) patients in Saudi Arabia

**DOI:** 10.1186/s40545-023-00589-2

**Published:** 2023-07-14

**Authors:** Ghada Ben Salah, Maryam Farooqui, Mohammed Salem Alshammari, Abir Elghazali, Lamyaa Kassem, Nada Ibrahim, Imen Ben Abdelmalek, Muhammad Kamran Rasheed

**Affiliations:** 1 Department of Pharmacology and Toxicology, Unaizah College of Pharmacy, Qassim University, Buraydah, 52571 Saudi Arabia; 2 Department of Pharmacy Practice, Unaizah College of Pharmacy, Qassim University, Buraydah, 52571 Saudi Arabia; 3 Department of Biology, College of Science, Qassim University, Buraydah, 52571 Saudi Arabia; 4 Department of Pharmacy Practice, College of Pharmacy, Qassim University, Buraydah, 52571 Saudi Arabia

**Keywords:** Complementary and alternative medicines, Chronic kidney disease, Treatment, Chronic disease, Cross-sectional studies, Health surveys, Public health, Adverse event, Saudi Arabia

## Abstract

**Introduction:**

Despite the paucity of scientific evidence, CAM is widely used for the prevention and treatment of illness among patients with chronic kidney disease, including end-stage renal disease and kidney transplant recipients. It is evident that the irrational use of CAM among CKD patients and its non-disclosure to healthcare providers could lead to adverse drug events. Hence, the current study was conducted to evaluate the prevalence, types, and non-disclosure of CAM use among CKD patients and kidney transplant recipients in Saudi Arabia.

**Methods:**

A cross-sectional study was conducted on 170 CKD patients (121 with stages 3 and 4, two with stage 5 and on hemodialysis, and 47 kidney transplant recipients). Face-to-face questionnaire-based interviews were conducted employing a convenience sampling technique. The study outcomes were the prevalence of CAM, types of CAM use, monthly expenditure on CAM, the source of information about CAM, and CAM disclosure to healthcare providers. A *p*-value of < 0.05 was considered significant.

**Results:**

The study found that out of 170, 60 (35.3%) CKD patients use CAM. The most used CAM was Acacia gum (49, 81.6%) followed by spiritual therapies (34, 56.6%). Female CKD patients had higher use of CAM compared to the male gender (*p* = 0.015). The monthly expenditures that most users (47, 78.3%) spent on CAM were less than 50 Saudi Riyals (SR). The study results also showed that 55% of CKD patients did not report their CAM use to their physicians. Furthermore, 46.6% of CAM users discontinue their use of CAM after observing no benefit.

**Conclusion:**

This study reported relatively high use of CAM among CKD patients in Saudi Arabia. The study found that most CKD patients use Acacia gum and spiritual therapies and do not disclose the use of CAM to healthcare professionals, which could lead to adverse drug events. Therefore, the study recommends that healthcare providers should inquire and provide evidence-based counselling about the use of CAM to CKD patients to prevent any adverse drug event or unwanted effect on the renal function of the patients.

## Introduction

Chronic kidney disease (CKD) is a non-communicable disease characterized by a persistent abnormality in the structure or function of kidneys for more than 3 months, with adverse complications CKD is ranged from Stage 1 (mild) to Stage 5 which is an end-stage renal disease (ESRD) and requires dialysis or a renal transplant surgery [1–3]. Recent data suggest that 9.1 to 13.4% of the worldwide population has CKD and its prevalence is increasing in the world, partly due to risk factors such as obesity and diabetes mellitus [4, 5].

In Saudi Arabia, CKD has been recognized as a major health problem in recent decades due to the growing incidence and prevalence of ESRD among the Saudi population [6]. An epidemiological study conducted in Saudi Arabia found the overall prevalence of CKD at 5.7% [7]. Saudi Arabia has seen a 25.1% increase in CKD patients in a span of 10 years with two million cases of CKD and 3818 deaths reported in the year 2017 alone [8]. This increase in CKD may be attributed to the rising number of hypertension, diabetes mellitus and obesity in the country [9, 10].

Complementary and Alternative medicine (CAM) is defined as a group of diverse medical and healthcare systems, practices, and products that are not generally considered to be a part of conventional medicine [11]. Globally, CAM use among patients with CKD and ESRD varies in the general population, accounting for 10–40% in European countries, 40–60% in USA, 49% in Australia, 46% in Malaysia, 75% in African countries, 64.8–86.6% in Iran, and up to 21.6–90% in Saudi Arabia [12–18]. Henceforth, because of immense financial and psychosocial burden, which affects the health-related quality of life over time, many CKD patients shifts to CAM practices to manage the symptoms of the CKD [19, 20]. The CAM used among CKD patients in Saudi Arabia is widespread, studies reported that 50–60% of CKD patients uses CAM, of whom 88.4% uses herbal products, while another findings, reported that 50.1% of hemodialysis patients (ESRD) uses CAM in Saudi Arabia [18, 21, 22]. It has been observed that the practices of CAM in Saudi Arabia are usually related to the religious beliefs of the consumers which include to seek spiritual therapies like reading Holy Quran, Zamzam water drinking and using honey, black seed, and myrrh [21, 23]. In addition to Alhijama (cupping therapy) is also considered to be a part of the prophetic medicine (authenticated by the Book of Sunnah) [24, 25].

The safety and efficacy of CAM, especially herbal remedies in CKD patients and kidney transplant recipients have not been established by evidence and could lead to adverse drug events, nephrotoxicity, and significant drug–nutrient interaction with immunosuppressive and other medications, especially for renal allograft patients [26–28]. Henceforth this study was planned and conducted to evaluate the prevalence, types and most common CAM use among CKD patients and kidney transplant recipients in the Qassim province of Saudi Arabia. In addition, the study also investigated the source of information about CAM use, monthly expenditure on CAM and the if the CKD patients and kidney transplant recipients disclose their CAM use to healthcare providers.

## Methods

### Study design and setting

A cross-sectional study was conducted among patients with CKD between December 2018 and January 2019 at the outpatient clinics and dialysis Centre (Daiverum) in King Saud Hospital (KSH), Qassim, Saudi Arabia. KSH Hospital is one of the biggest governmental referral hospitals for CKD patients in the region and caters for all types of CKD cases including hemodialysis and kidney transplant on a regular basis. This study was approved by the Qassim Bioethics Research Committee (Approval no: 2018-11-29).

### Sample size

The population size of CKD patients at KSH was estimated to be 400. Using a confidence interval of 95%, a standard deviation of 0.5, and a margin of error of 5%, the minimum required sample size came out to be 197 study participants. During the two months of the data collection period, 191 CKD patients were approached, and 170 consented to participate in the study, giving a response rate of 89%.

### Study tool

To collect data, we used a questionnaire adopted from a previous study about CAM use among cancer patients after getting official permission from the authors [28]. The questionnaire was in English language and consists of 17 items subdivided into four sections. The first section captured the demographics (age, sex, marital status, educational level, current job, place of living, and family monthly income). The second section included the disease characteristics (CKD stages) of the study participants, while the third part of the questionnaire documents the details of the types of CAM use, its frequency, reasons for using, and sources of information on using CAM. The fourth section documents the disclosure of CAM use to the physician and reasons if there is a non-disclosure. The questionnaire is modified by the researchers to suit local ethical and cultural requirement of Saudi Arabia. Since Arabic is the official language among the general population of Saudi Arabia, the questionnaire was translated into Arabic language using the forward-backwards translation technique [29]. The forward translation was done by three native Arabic speakers. The translated draft was revised by a nephrologist in the KSH, a bilingual social worker, and 3 CKD patients for the face and content validity. Some modifications were made to the questionnaire after the revision. This step was followed by a backward translation of the Arabic version of the questionnaire into the English language by a bilingual clinical pharmacist who was not aware of the original questionnaire. Finally, the backward translated version was compared carefully with the original English version to establish the content validity of the original version.

### Sampling strategy and data collection

Convenience sampling was adopted to select CKD patients in this study. Patients older than 16 years, diagnosed as having CKD at any stage, receiving hemodialysis or peritoneal dialysis and recipients of a kidney transplant were included in the study. Patients who had mental or psychological limitations that made them incapable of responding to the survey were excluded. During the two months of the data collection period (Dec 2018–Jan 2019), patients were approached during their admission to the nephrology ward of KSH. Prior to their voluntary participation, an explanatory statement detailing the objectives of the study was given to them and written consent was obtained. Patients were asked the questions by reading questions to them from the researchers. Answers from the patients were recorded immediately and another researcher made sure that the correct answers were recorded.

### Data analysis

Descriptive and inferential statistical analyses were performed using the Statistical Software Package for the Social Sciences version 23 for Windows (Chicago, IL). For descriptive statistics, we computed frequencies, and percentages for categorical variables. Pearson Chi-square test or Fisher’s exact test was used for the comparative analysis of patient’s demographic data and survey results. *p* < 0.05 was considered significant.

## Results

### Demographics and disease characteristics of CKD patients and their CAM use

A total of 170 CKD patients participated in this survey. The socio-demographics and disease characteristics of the study population and their CAM use are shown in Table 1. Out of 170 patients, 47 were at CKD stages 3–4, 121 were at CKD stage 5 hemodialysis, and 2 were CKD-kidney transplant recipients. The majority of the patients were females (54%), aged more than 61 years (37.1%), and married (65.3%). Out of 170 CKD patients, 60 (35.3%) reported using at least one type of CAM since their CKD diagnosis. The subgroup analysis revealed that CAM use was the highest among stage 5 hemodialysis patients (76.6%), followed by patients with CKD Stages 3–4 (21.6%), and CKD-transplant recipients (1.8%). However, no statistical association was found between CKD severity levels and CAM use (*p* = 0.35). Among all the socio-demographic variables, female gender (*p* = 0.015) was significantly associated with CAM use (Table 1).

**Table 1:** Demographic distribution and disease characteristics of CKD patients and their CAM use

Characteristics	No. (%) *N* = 170	CAM use		*p* value
		No. (%) No = 110	No. (%) Yes = 60	
*Age*				
16–20	6 (3.5)	5 (4.5)	1 (1.7)	
21–30	12 (7.1)	9 (8.2)	3 (5.0)	
31–40	16 (9.4)	10 (9.1)	6 (10.0)	
41–50	33 (19.4)	17 (15.5)	16 (26.7)	0.137
51–60	40 (23.5)	22 (20.0)	18 (30.0)	
> 60	63 (37.1)	47 (42.7)	16 (26.7)	
*Gender*				
Male	78 (45.9)	58 (52.7)	20 (33.3)	0.015*
Female	92 (54.1)	52 (47.3)	40 (66.7)	
*Marital status*				
Unmarried	21 (12.4)	14 (12.7)	7 (11.7)	
Married	111 (653)	70 (63.6)	41 (68.3)	
Divorced	10 (5.9)	6 (5.5)	4 (6.7)	0.845
Widowed	28 (16.5)	20 (18.2)	8 (13.3)	
*Education level*				
Primary education	35 (20.6)	26 (23.6)	9 (15.0)	
Middle school	26 (15.3)	12 (10.9)	14 (23.3)	
Secondary education	33 (19.4)	24 (21.8)	9 (15.0)	
Diploma/matriculation	13 (7.6)	5 (4.5)	8 (13.3)	
University degree	18 (10.6)	12 (10.9)	6 (10.0)	0.127
Post-graduate degree	1 (0.6)	1 (0.9)	0 (0.0)	
Never go to school	42 (24.7)	29 (26.4)	13 (21.7)	
Others^¶^	2 (1.2)	1 (0.9)	1 (1.7)	
*Employment status*				
Unemployed	39 (22.9)	28 (25.5)	11 (18.3)	
Employed	26 (15.3)	16 (14.5)	10 (16.7)	
Retired	44 (25.9)	34 (30.9)	10 (16.7)	
Homemaker	48(28.2)	25 (22.7)	23 (38.3)	0.121
Student	8 (4.7)	5 (4.5)	3 (5.0)	
Others ǂ	5 (2.9)	2 (1.8)	3 (5.0)	
*Monthly income*				
< 5000	109 (64.1)	72 (65.5)	37 (61.7)	
5000–10,000	41 (24.1)	23 (20.9)	18 (30.0)	0.310
> 10,000	20 (11.8)	15 (13.6)	5 (8.3)	
*CKD severity*				
CKD stages 3–4	121 (71.2)	75 (68.1)	46 (76.6)	
Stage 5 hemodialysis	2 (1.2)	1 (0.9)	1 (1.7)	0.355
CKD-transplant	47 (27.6)	34 (30.9)	13 (21.7)	

*Statistically significant *p* value was calculated using Chi-square test. ^¶^Incomplete primary education ^ǂ^Self employed

### Types of complementary and alternative medicinal therapies used by CKD patients

Among the 170 participants, 60 (35.3%) reported using CAM modalities. The most used modalities were herbal therapies (28.2%), followed by spiritual therapies, such as Quran recitation and the holy water of Zamzam (20%). Other less popular CAM practices were, honey (1.2%), and multivitamins/supplements (0.6%), as illustrated in Table 2.

**Table 2:** Types of CAM use among CKD patients

Type of CAM	*N* = 60 (35.3%)
Acacia gum	49 (28.8%)
Spiritual therapies (prayers, charities, Quran recitations and holy water of Zamzam)	34 (20%)
Honey	2 (1.2%)
Multivitamins and other supplements	1 (0.6%)

Total percentage may be not 100% due to the choice given for multiple responses

### Monthly expenditures and sources of recommendation for different types of CAM therapies used by the CKD patients

The monthly expenditure that most users (47, 27.6%) spent on CAM was less than Saudi Riyal (SR) 50. About 16% of the study participants rely on advice from family members or friends as a source of information about CAM. The second most reliable source of information was social media (Facebook, Instagram, Twitter) (12%), followed by their own decision (10%). The sources of information on the CAM are summarized in Table 3.

**Table 3:** Monthly expenditure and source of recommendation of CAM use among CKD patients

Monthly expenditure of CAM in SAR	*N* (%)
*Total amount in Saudi Riyal (SR)*	
< 50 SR	47 (27.6%)
51–100 SR	3 (1.8%)
101–200 SR	2 (1.2%)
201–300 SR	1 (0.6%)
> 300 SR	7 (4.1%)
*Sources of recommendation about CAM*^*a*^	
By family members or friends	28 (16.5%)
Social medias and Internet	21 (12.4%)
Your own free will	17 (10.0%)
By the physicians/pharmacists/nurses	12 (7.1%)
By other CKD patients	5 (2.9%)
Newspapers and televisions	1 (0.6%)
Others	1 (0.6%)

^a^Total percentage may be not 100% due to the choice given for multiple responses; SAR: 1 Saudi Riyal = USD 0.27

### CAM monthly expenditure and disclosure to doctors

The majority of the participants reported to spend a very minimal amount on CAM which was less than SR 50 (Table 4). Only 45% of the CKD patients who uses CAM, disclosed the CAM use to their healthcare professionals. The majority of the physicians (13, 48%) were supportive of the CAM use by their CKD patients, when the patients disclosed to them. In contrast, the majority of patients, who did not disclose their CAM use to physicians, mentioned that it’s not important to disclose.

**Table 4:** Disclosure of CAM use to physicians by CKD patients

Variables	*N *(%)
*Did your doctor/nephrologist ask about your CAM use? (N = 60)*	
Yes	19 (31.7%)
No	39 (65%)
Not sure	2 (3.3%)
*Did you disclose your CAM use to your doctors/ nephrologist?*	
Yes	27 (45%)
No	32 (53.3%)
Not sure	1 (1.6%)
*How did your doctor respond upon your CAM disclosure? (N* = *27)*	
Agree of CAM use	13 (48.1%)
Disagree of CAM use	12 (44.4%)
Neutral about CAM use	2 (3.3%)
*Why did you not mention it to your doctor? (N* = *32)*	
I thought it is not important for my doctor to know	16 (50%)
I thought my doctor has little or no knowledge about CAM	3 (9.4%)
I thought the doctor would disagree	1 (3.1%)
I thought my doctor will stop my treatment	0
Doctors never ask about CAM so I did not tell	12 (37.5%)

## Discussion

Studies on the use of CAM in CKD populations are growing worldwide due to increasing reports on the harmful effects of certain herbal products on kidneys [30, 31]. Nevertheless, in Saudi Arabia, most of the published studies have looked at the CAM use in Saudi patients with various chronic diseases [18, 21].

In this study, the overall prevalence of CAM use among CKD patients in the Qassim region of Saudi Arabia was 35%. This is in contrast to a previous study conducted in other parts of Saudi Arabia, who reported a higher prevalence of CAM use among CKD (54.9%) and ESRD patients (50.1%) [21, 23]. In addition, CAM use among the CKD patients in other parts of the world also showed a high prevalence, including those in Malaysia (63.6%), Palestine (64.4%), Egypt (52%), United States (61%), and in India (66.3%) [14, 32–35].

In the current study, no association was found between the use of the CAM and socio-demographic and disease characteristics, with the exceptions of a female gender (*p* = 0.015). These findings are similar to previously reported figures among Saudi patients with ESRD which found that the use of the CAM was significantly higher among women than men [23]. These findings is in contrast to the previously published literature that reported a significant influence of patients’ demographic characteristics and CAM practice among CKD patients in other parts of the world, including Arab countries in the Middle East [26, 27, 32, 34]. The difference in the results between various studies about CAM practice may be related to the differences in the study sample, the geographical setting of these studies, or the fact that patients were unwilling to report their CAM use to the healthcare providers. It has been suggested previously that CAM use is more common among the well-educated and wealthy [36]. This is somewhat not true in our study findings, where most of the CAM users were homemakers and who never attended school. Most of the CAM users reported spending a minimal amount on CAM (less than SR 50). A possible reason could be that the type of CAM preferred by the majority of the participants was prayers and spiritual therapies, which are mostly self-practiced without any monetary expenditures. In addition, as the majority of CAM users were homemakers with no fixed monthly income, a minimal expenditure on CAM use is justifiable. The study results also showed that patients at the early stages of CKD showed more interest in using CAM than those at the advanced stages.

The popularity of traditional Arab medicines is strongly influenced by religious, cultural, and social factors, rather than evidence-based medicines or recommendations from the healthcare professionals [18]. Similarly, the findings of this study reported that herbal supplements and recitation of the Quran were found to be the most popular CAM therapies among the study population. Similar findings were reported in previous CKD studies, in other Arab and countries, such as Palestine, and Egypt [32, 33].

The most common herb used by the study participants was Arabic gum (AG) both alone and in combination with other CAM modalities like spiritual therapy and multivitamins. The AG is a complex polysaccharide of natural plant origin which is a highly fermentable dietary fiber with proven prebiotic properties [37]. Additionally, supplementation of CKD patients' diet with 10–40 g/day of AG, could significantly reduce inflammatory markers in pre-dialysis CKD, and could have a positive impact on the long-term morbidity and mortality of CKD patients [38, 39].

This study’s findings reported that the majority of CAM users did not disclose their CAM practices to their physicians. Similar findings were reported by previous studies conducted in different parts of the world, where the study participants hesitated to share any information regarding CAM use with their physicians or healthcare providers [14, 21, 32, 40]. In our study, the main reason for non-disclosure of CAM use, was the lack of enquiry about CAM use from healthcare providers, which was also reported by previous studies [41, 42]. Therefore, communication between healthcare providers and patients is crucial to protect CKD patients from the suspected adverse effects of CAM. Various studies documented that healthcare providers should initiate the discussion and encourage their patients to talk about CAM use to avoid potential harm to the kidneys [43, 44]. The role of pharmacists to educate CKD patients and other healthcare professionals about the safe and effective use of CAM and to highlight the potential toxic effects of CAM has been established [45]. Hence, pharmacists in Saudi Arabia could be utilized to educate CKD patients about the CAM use [46, 47].

There are a few limitations to this study. First, the study participants were from a single hospital in the Qassim region of Saudi Arabia, hence the sample size was relatively low and may not be representative of all CKD patients in Saudi Arabia Second, the lack of privacy during asking questions to CKD patients may have also affected the results, hence, patients might have not felt comfortable disclosing certain information about the CAM use.

## Conclusion

In conclusion, the prevalence of CAM use is relatively low among the selected CKD patients. Most of the CKD patients use Arabica gum as CAM, followed by spiritual therapies of reading Quran or consuming the holy water of Zamzam. The majority of the CKD patients did not disclose CAM use to their physicians because they were either not asked about it. Thus, it is critical for healthcare professionals to be more vigilant regarding the use of CAM among CKD patients and it is recommended that healthcare professionals should ask the patient about the CAM practice at every stage of their disease and monitor patients for any adverse effects of CAM. Therefore, there is a need for continuing professional development programs for healthcare providers about CAM modalities, their potential benefits and harm.

## Data Availability

The prospective data used to support the findings of this study are available from one of the corresponding authors (Dr Maryam Farooqui) upon request.
